# A Novel Fractional Brownian Dynamics Method for Simulating the Dynamics of Confined Bottle-Brush Polymers in Viscoelastic Solution

**DOI:** 10.3390/polym16040524

**Published:** 2024-02-15

**Authors:** Shi Yu, Ruizhi Chu, Guoguang Wu, Xianliang Meng

**Affiliations:** 1 Department of Chemical Engineering, China University of Mining & Technology, Xuzhou 221116, China; rzchu@cumt.edu.cn (R.C.); ggwu@cumt.edu.cn (G.W.); meng27@cumt.edu.cn (X.M.); 2 Key Laboratory of Coal-Based CO_2_ Capture and Geological Storage, China University of Mining & Technology, Xuzhou 221116, China

**Keywords:** fractional Brownian motion, Brownian dynamics (BD) simulation, bottle-brush polymers

## Abstract

In crowded fluids, polymer segments can exhibit anomalous subdiffusion due to the viscoelasticity of the surrounding environment. Previous single-particle tracking experiments revealed that such anomalous diffusion in complex fluids (e.g., in bacterial cytoplasm) can be described by fractional Brownian motion (fBm). To investigate how the viscoelastic media affects the diffusive behaviors of polymer segments without resolving single crowders, we developed a novel fractional Brownian dynamics method to simulate the dynamics of polymers under confinement. In this work, instead of using Gaussian random numbers (“white Gaussian noise”) to model the Brownian force as in the standard Brownian dynamics simulations, we introduce fractional Gaussian noise (fGn) in our homemade fractional Brownian dynamics simulation code to investigate the anomalous diffusion of polymer segments by using a simple “bottle-brush”-type polymer model. The experimental results of the velocity autocorrelation function and the exponent that characterizes the subdiffusion of the confined polymer segments can be reproduced by this simple polymer model in combination with fractional Gaussian noise (fGn), which mimics the viscoelastic media.

## 1. Introduction

Macromolecular diffusion in crowded and heterogeneous cytoplasm is essential in many intracellular processes, such as metabolism, signal transduction, transcription, and translation [1,2]. Many imaging experiments demonstrate that chromosomal loci and cytoplasmic particles exhibit anomalous subdiffusion in living cells [3,4,5,6]. Thus, it is of great interest to investigate the dynamics of the genome and cytosol in bacterial cells both experimentally and theoretically to elucidate the origin of this anomalous diffusion. Due to the temporal and spatial resolution limit of single-molecule tracking, studying the interactions among various biological macromolecules in real time is extremely challenging. Therefore, many researchers examined how the dynamics of bacterial genomes and cytosol are altered by different treatments to bypass the resolution limit. Parry et al. [7] revealed that the bacterial cytoplasm is fluidized by ATP-dependent cellular metabolic activities, which agrees with Weber’s observation [3] that the motions of genomic loci can be suppressed by the depletion of ATP in *E. coli* bacterial cells. Okumus et al. [8] showed that the diffusion of cytoplasmic proteins can be slowed down significantly by compressive forces exerted onto *E. coli* cells. Moreover, Wlodarski et al. [9,10] investigated the effects of sublethal antibiotic treatments on the dynamics of both genome and cytosol in *E. coli*. Although bacterial genome and cytosol responses to external perturbation can provide insight into intracellular dynamics, a detailed molecular model that resolves the heterogeneous and crowded cytoplasm is still necessary to fully understand the subdiffusive behaviors of various biological macromolecules.

All-atom molecular models of cytoplasm [11], DNA [12], and cell membranes [13] can characterize the molecular-level interactions in cells with an unprecedented resolution. However, the all-atom molecular dynamics method is computationally expensive, which drives the development of widely used coarse-grained models of biological macromolecules, such as Martini force field [14], the oxDNA model [15], and Shinoda’s model of lipids [16]. Although molecular dynamics simulations can be sped up dramatically with those coarse-grained force fields due to the reduction in the number of degrees of freedom, it is still difficult to reproduce the anomalous diffusion by using those coarse-grained models since cytoplasmic particles/proteins and chromosomal loci can scan a large fraction of bacterial cells by performing subdiffusion [3,7,8]. Therefore, extensive research has focused on the simulation of cytoplasmic anomalous diffusion by using mesoscopic scale models [17]. Ando et al. [18] and Hasnain et al. [19] used spherical particles or several connected beads of different sizes to simulate bacterial cytoplasm and demonstrated that the anomalous diffusion can be attributed largely to the crowding effect. Słyk et al. [20] compared the effects of hard crowders on cytoplasmic diffusion to that of soft crowders, describing both hard and soft crowders by spherical particles. Swain et al. [21] studied the effects of crowding and confinement on the dynamics of bacterial chromosomes by using a “bottle-brush”-type polymer model [22,23]. Although the dynamics of the loop formation of a nucleoid can be very complex due to topological entanglements as well as polydispersity in loop sizes, the helical shape with a ∼1 μm pitch of bacterial chromosomes in rod-shaped bacteria like *E. coli* can be predicted by a minimal structured polymer model [22,23,24]. In addition, the subdiffusion of chromosomal loci due to DNA bridging was also simulated by S. Subramanian and S. M. Murray [25] by using the bond fluctuation method.

To further reduce the degrees of freedom of the system, different types of random walks (e.g., obstructed diffusion, fractional Brownian motion, and continuous time random walks) in which cytosol is modeled implicitly were proposed to explain the anomalous diffusion of tracers in cells [26]. Although anomalous subdiffusion can be generated with obstructed diffusion (OD) [27] as well as a continuous time random walk (CTRW) [28,29], the subdiffusion in a cell is likely a result of the intrinsic viscoelasticity of cytoplasm since the cytoplasmic particle velocity is anticorrelated for short time lags, which is contradicting to OD or CTRW models [3,29]. Such an anticorrelation of particle displacements can be successfully reproduced by fractional Brownian motion (fBm) [28,29,30] because fractional Brownian motion is a highly correlated/anticorrelated process [31]. On the other hand, cytoplasmic subdiffusion has a non-Gaussian distribution of increments [6,7], which is similar to the so-called “Brownian yet non-Gaussian” dynamics found in a growing number of biological, soft, and active matter systems [32]. Many researchers [32,33,34] adopted the diffusing or random diffusivity models to explain this non-Gaussian behavior. The predictions of the diffusing diffusivity model qualitatively agree with the experimental observations of the non-Gaussian behaviors of cytoplasmic tracers since the effects of the heterogeneous environment on subdiffusive particles have been included in such models [35]. Baldovin et al. [36] argued that such diffusing diffusivity models work at a mesoscopic scale, which is not directly related to the underlying molecular processes. So, they [36] proposed a specific example of the dynamical behaviors of macromolecules during simple polymerization processes, which can bridge the gap between diffusing diffusivity models and molecular-scale processes. In addition, metabolic activities that drive the system out of the equilibrium state might also contribute to the long tails of the displacement distributions of the particles performing non-Gaussian diffusion. Such nonequilibrium activities were simulated by active forces in Fodor’s work [37].

As discussed above, the crowding effects, the viscoelasticity of the cytoplasm, the DNA bridging, the heterogeneous environment causing diffusing diffusivity, and even the nonequilibrium activity have been considered separately in previous theoretical and numerical works. However, the non-Gaussian subdiffusion of chromosomal loci and cytoplasmic particles has yet to be fully reproduced since Markovian processes (as in simple diffusing diffusivity models) cannot describe the anticorrelation of particle displacements and fBm cannot simulate non-Gaussian behaviors as fBm is a mean-zero Gaussian process [30,31]. Moreover, the application of the fractional Brownian motion model to viscoelastic-type subdiffusion was often limited to single tracers [29]. Therefore, in this manuscript, we consider a particular model that combines a “bottle-brush”-type polymer model [22] and fractional Brownian motion to explore to what extent the anomalous diffusion of polymer segments is affected by the dynamical structure of the bottle-brush polymers and the viscoelasticity of the surrounding environment. The effects of viscoelastic crowded media on the polymer segments were included in the fractional Brownian motion increments for each polymer bead, allowing us to consider the crowders implicitly to reduce the degrees of freedom of the whole system. Another advantage of introducing fractional Brownian motion to bottle-brush polymers simulations is that the nonequilibrium activities that take place in vivo can be modeled as accelerated Brownian dynamics [38] with a large Hurst parameter (H∈(0.5,1)) for fractional Brownian motion. Although the heterogeneity of the surrounding environment might be introduced to this model by random variations in the polymer bead size in an overdamped regime, here, in this work, we focus on the simulation of the “non-Brownian yet Gaussian” diffusion of bottle-brush polymers by using a polymer model composed of fractional diffusive monomers.

## 2. Models and Simulation Methods

### 2.1. Force Fields

**Figure 1:**
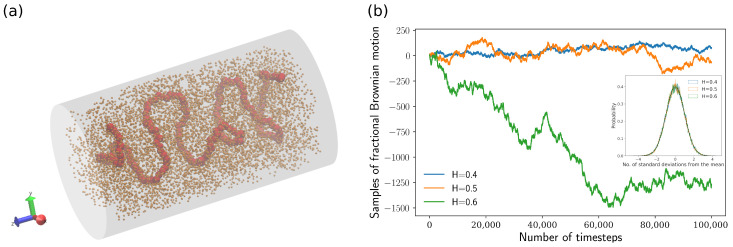
(**a**) Snapshot of “bottle-brush” polymer model under cylindrical confinement. The backbone chain and side loops are represented by red and orange beads, respectively. (**b**) Samples of fractional Brownian motion (fBm) for H=0.4, H=0.5, and H=0.6. (Inset) histogram of fractional Gaussian noise (fGn), i.e., fBm increments for H=0.4, H=0.5, and H=0.6. The black dashed curve represents the normal distribution.

In this manuscript, the confined bottle-brush polymer was modeled by a linear backbone chain of 200 beads connected by springs [22]. Moreover, 40-bead side loops were attached to every bead of the backbone chain as described by D. Chaudhuri and B. M. Mulder [22,23]. As discussed as follows, to study to what extent the diffusive behaviors of polymer segments are affected by polymer stiffness, both FENE (finitely extensible nonlinear elastic) bond energy [21] and harmonic bond energy [22] were used to model the springs connecting the polymer beads. The FENE bond energy is given by

(1) $$U_{FENE}=-\frac{1}{2}KR^{2}ln\left[1-{\left(\frac{d_{i}-\sigma }{R}\right)}^{2}\right]$$

where $d_{i}=\left|d_{i}\right|$ is the distance between consecutive beads, i.e., the bond length ($d_{i}=r_{i+1}-r_{i}$ is the bond vector); $K=30\epsilon /\sigma^{2}$; R=1.5σ; and ϵ=1.0 and σ=1.0 are the unit of energy and unit of length in our simulations, respectively. The harmonic bond energy is as follows:

(2) $$U_{harmonic}=\frac{1}{2}k_{bond}{\left(d_{i}-\sigma \right)}^{2}$$

where $k_{bond}=100\epsilon /\sigma^{2}$ is the harmonic bond energy coefficient, $d_{i}$ is the distance between consecutive beads, and σ is the equilibrium bond length and the unit of length. To avoid the overlapping of polymer beads, the excluded volume interactions between the nonbonded beads are modeled by the Weeks–Chandler–Andersen (WCA) potential [22], which is given by

(3) $$U_{WCA}\left(r_{ij}\right)=\left\{\begin{array}{c} 4\epsilon \left[{\left(\frac{\sigma }{r_{ij}}\right)}^{12}-{\left(\frac{\sigma }{r_{ij}}\right)}^{6}+\frac{1}{4}\right],\;r_{ij}\leq 2^{1/6}\sigma \\ 0,\;\;\;\;\;\;r_{ij}>2^{1/6}\sigma \end{array}\right.$$

where $r_{ij}=\left|r_{ij}\right|$ is the distance between the $i^{th}$ bead and $j^{th}$ bead; the cutoff distance for the WCA potential is $2^{1/6}\sigma$. The confining walls (grey surfaces, as shown in Figure 1a) for all polymer beads are modeled through the $U_{wall}$ [22], which is given by

(4) $$U_{wall}\left(r_{iw}\right)=\left\{\begin{array}{c} 2\pi \epsilon \left[\frac{2}{5}{\left(\frac{\sigma }{r_{iw}}\right)}^{10}-{\left(\frac{\sigma }{r_{iw}}\right)}^{4}+\frac{3}{5}\right],\;r_{iw}\leq \sigma \\ 0,\;\;\;\;\;\;r_{iw}>\sigma \end{array}\right.$$

where $r_{iw}$ is the distance between the $i^{th}$ bead and the wall. Some polymer beads may be close to (i.e., $r_{iw}<\sigma$) both the cylindrical side surface and the top (or bottom) surface, and then $U_{wall}$ potentials for these two surfaces are computed separately. As shown in Figure 1a, the length of the cylindrical confinement in the *z*-axis direction is 50.75 σ, and the radius of the cylinder is 14.75 σ.

### 2.2. Fractional Brownian Dynamics Simulation

We update the bead positions based on the following equation [39]:

(5) $$r^{\left(n+1\right)}=r^{\left(n\right)}+\frac{\Delta t}{6\pi \eta a_{bead}}F^{\left(n\right)}+\sqrt{\frac{2k_{B}T\Delta t}{6\pi \eta a_{bead}}}\xi^{\left(n\right)}$$

where $r^{\left(n+1\right)}$ and $r^{\left(n\right)}$ are the bead positions at time step n+1 and *n*, respectively; Δt is the time step; $F^{\left(n\right)}$ is the total non-Brownian force on the bead at time step *n*; and $a_{bead}=\frac{1}{2}\sigma$ is the radius of the beads. Here we take $\eta =\frac{200}{3\pi \sigma }$ to be the cytoplasm viscosity. We use reduced units for our simulations where $k_{B}T=1$. We assume that all the beads of the polymer model perform fractional Brownian motion; that is, bead positions can be defined by the stochastic representation [40] described as follows if the total non-Brownian force is zero:

(6) $$B_{H}\left(t\right):=\frac{1}{\Gamma \left(H+\frac{1}{2}\right)}\left(\int_{-\infty }^{0}\left[{\left(t-s\right)}^{H-\frac{1}{2}}-{\left(-s\right)}^{H-\frac{1}{2}}\right]dB\left(s\right)+\int_{0}^{t}{\left(t-s\right)}^{H-\frac{1}{2}}dB\left(s\right)\right)$$

where Γ is the Gamma function $\Gamma \left(\alpha \right):=\int_{0}^{\infty }x^{\alpha -1}e^{-x}dx$, H∈(0,1) is the Hurst parameter, and the integrator *B* is an ordinary Brownian motion. Note that the fractional Brownian motion $B_{H}$ is reduced to an ordinary Brownian motion by taking $H=\frac{1}{2}$ in Equation (6). Three example tracks of one-dimensional (1D) fractional Brownian motion for different Hurst parameters are depicted in Figure 1b. To ensure that polymer beads perform fractional Brownian motion if the total non-Brownian force is absent, $\xi^{\left(n\right)}$ in Equation (5) was chosen as a vector of fractional Brownian motion increments, i.e., fractional Gaussian noise (fGn), at time step *n*. The three components of the vector $\xi^{\left(n\right)}$ in the three-dimensional Cartesian coordinate system are denoted by $X_{1}^{\left(n\right)}$, $X_{2}^{\left(n\right)}$, and $X_{3}^{\left(n\right)}$:

(7) $$X_{i}^{\left(n\right)}:=B_{H}\left(n+1\right)-B_{H}\left(n\right)\;\left(i=1,2,3\right)$$

Each $X_{i}^{\left(n\right)}$ has a standard normal distribution [41], as shown in the inset of Figure 1b. The autocovariance function γ(·) of $X_{i}^{\left(n\right)}$ is given by the following equation [41]:

(8) $$\gamma \left(k\right)=EX_{i}^{\left(n+k\right)}X_{i}^{\left(n\right)}=\frac{1}{2}\left[{\left|k-1\right|}^{2H}-{2|k|}^{2H}+{\left|k+1\right|}^{2H}\right]$$

for a discrete-time random process (k,n∈Z). For H=0.5, Equation (8) is reduced to γ(k)=0 for k≠0, where an ordinary Brownian motion is recovered. Note that fractional Brownian motion is not a Markov process based on Equation (6), and there is (in general) no independence in fractional Gaussian noises $X_{i}^{\left(n\right)}$ (as demonstrated in Equation (8)). However, for simplicity, the 3 components of vector $\xi^{\left(n\right)}$ are extracted from three independent realizations of fractional Gaussian noises, which means $EX_{i}^{\left(n+k\right)}X_{j}^{\left(n\right)}=0$ for i≠j. And in this manuscript, the fractional Gaussian noise $X_{i}^{\left(n\right)}$ was generated by the fBm-0.3.0 package programmed by Christopher Flynn by using the Davies Harte method [42].

The dynamics of “bottle-brush”-type polymers in cylindrical confinement were investigated systematically. The initial configurations of our simulation systems were prepared by using $2\times 10^{6}$ time steps with long standard Brownian dynamics (BD) equilibration in an overdamped regime, which can be achieved simply by setting H=0.5 for $\xi^{\left(n\right)}$ in Equation (5). Then, fractional Brownian dynamics simulations (based on Equation (5)) of $1\times 10^{7}$ time steps were carried out for various Hurst parameters. Only the last $8\times 10^{6}$ time steps’ simulation results were used for further analysis. Moreover, the time step Δt was set to 0.01 for all our simulations unless otherwise stated.

## 3. Results and Discussion

### 3.1. Potential Energy vs. Time for Standard and Fractional Brownian Dynamics Simulations

**Figure 2:**
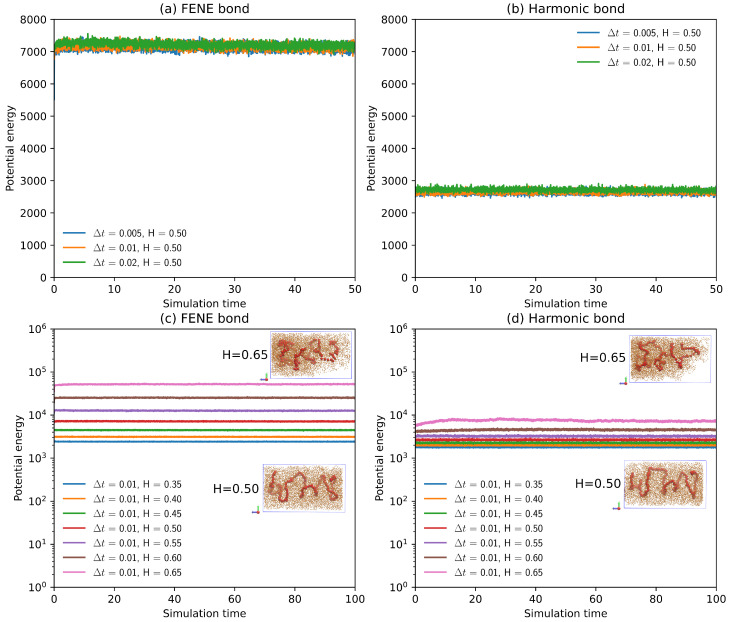
(**a**) The potential energy of a polymer model with FENE bonds as a function of simulation time for different time steps Δt, obtained by standard Brownian dynamics simulation (H=0.5) in an overdamped regime (Equation (5)). (**b**) The potential energy of a polymer model with harmonic bonds as a function of simulation time for different time steps Δt. (**c**) The potential energy of a polymer model with FENE bonds as a function of simulation time for different Hurst parameters, obtained by fractional Brownian dynamics simulation. (Inset) snapshots of polymer models in cylindrical confinement with H=0.65 and H=0.50. (**d**) The potential energy of a polymer model with harmonic bonds as a function of simulation time for different Hurst parameters by fractional Brownian dynamics simulation. (Inset) snapshots of polymer models for H=0.65 and H=0.50.

As described above, we use a simple “bottle-brush” polymer model in combination with fractional Gaussian noises to simulate the anomalous diffusive behaviors of polymer segments. As demonstrated in Figure 2a,b, the potential energy of the simulation system reaches a plateau rapidly for polymer models with both FENE bonds and harmonic bonds as Δt varies from 0.005 to 0.02. The potential energy curves in Figure 2a,b indicate that system equilibrium is obtained by a standard Brownian dynamics simulation since a fractional Brownian motion is reduced to an ordinary Brownian motion by setting the Hurst parameter *H* to 0.50. The potential energy of the polymer model with FENE bonds is much larger than that of the polymer model with harmonic bonds, which implies that the polymer model is much “stiffer” with FENE bonds.

To test the suitability of the fractional Brownian dynamics simulation, the influence of fractional Gaussian noises on the potential energy of the simulation system is examined by varying the Hurst parameter *H*. The potential energy curves for both the FENE and the harmonic bond models are plotted against simulation time for different *H*, as shown in Figure 2c,d. Again, a fractional Brownian dynamics simulation can rapidly achieve the equilibrium state, similar to a standard Brownian dynamics simulation. The potential energy increases for both FENE and harmonic bonds as the Hurst parameter *H* increases from 0.35 to 0.65. Note that fractional Brownian motion with H∈(0,0.5) is subdiffusive, while fractional Brownian motion with H∈(0.5,1) is an accelerated Brownian motion [38] or superdiffusive. As the Hurst parameter approaches 1.0, the fractional Brownian motion becomes a ballistic-like motion. As a result, although the bead positions are updated based on Equation (5), where the inertia of the polymer beads is neglected, the polymer beads act like they have random “momenta”, owing to the highly correlated fractional Gaussian noises for the large Hurst parameter H∈(0.5,1). Since the direction and magnitude of the correlated fGn changes less frequently than white noise for a large H∈(0.5,1), the correlated fGn works as an external random “force” or “impulse” exerted on the polymer beads, which drives the system out of equilibrium. Such random “impulses” greater than thermal fluctuations cause the increase in potential energy of the simulation system for large Hurst parameters, as seen in Figure 2c,d. As shown by the pink curve in Figure 2d, the time it takes to equilibrate the simulation system is much longer for H=0.65 than that for a smaller *H*, and the fluctuations in the potential energy are stronger for H=0.65 because the correlated fGn condenses the polymer beads for the large Hurst parameter. Snapshots of the condensed polymer model with H=0.65 are presented as the insets of Figure 2c,d.

**Figure 3:**
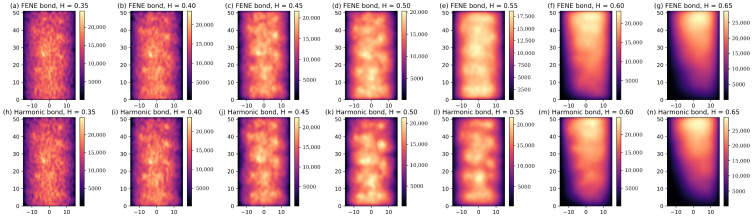
(**a**–**n**) Projected number density profiles in xz plane of polymer beads on polymer models with FENE and harmonic bonds for different Hurst parameters *H*.

Bacterial chromosomes are highly dynamic structures [43] whose center of mass is precisely located at the center of the cell volume [21]. To compare this observation [21] to our simulation results, the two-dimensional projected number densities of the polymer beads in the xz-plane for various Hurst parameters are plotted in Figure 3. As can be seen from Figure 3d,k, the projected number densities of the polymer beads, which are obtained by standard Brownian dynamics simulations (H=0.5) for the models with both FENE and harmonic bonds, exhibit shapes with grooves. These simulation results agree with the helical shape of this simple polymer model predicted by Langevin dynamics simulations [22]. As the Hurst parameter *H* decreases from 0.5 to 0.35, i.e., entering the subdiffusion regime, the projected number density of polymer beads becomes more evenly distributed across the whole cross-sectional area of the cylindrical confinement, which is demonstrated in Figure 3a–d,h–k. On the other hand, as *H* increases from 0.5 to 0.65, the polymer beads are close to one end of the cylindrical confinement, acting as though they are pushed by external forces, as shown in Figure 3d–g,h–n. So, the center of mass of all the polymer beads is no longer positioned at the center of the cell volume (Figure 3f,g,m,n). The condensation of the polymer beads for a large *H* increases the potential energy of the simulation system, mainly due to the excluded volume interactions among the polymer beads, as shown in Figure 2c,d. Although switching from harmonic bonds to FENE bonds increases the potential energy significantly, the effects of the bond types on the projected number density of the polymer beads are negligible, as demonstrated in Figure 3f,g,m,n.

### 3.2. Diffusion Coefficient and Exponent α

**Figure 4:**
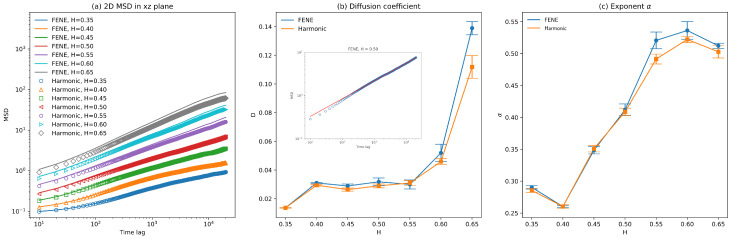
(**a**) Averaged 2D mean-squared displacement in xz plane as a function of time lag for polymer models with both FENE and harmonic bonds for different Hurst parameters. (**b**) Fitted diffusion coefficient as a function of Hurst parameter *H* for polymer models with FENE and harmonic bonds. (Inset) one example of fitting of averaged 2D MSD against time lag according to Equation (9). (**c**) Fitted exponent α as a function of Hurst parameter *H* for polymer models with FENE and harmonic bonds (the error bars are standard deviations).

Since fluorescence microscopy experiments record the two-dimensional diffusive behaviors of bacterial chromosomes in the focusing plane [3,4,5], to compare our fractional Brownian dynamics simulations directly to previous experimental observations, the diffusion coefficients, as well as the exponent α characterizing the anomalous diffusion, are obtained by fitting the 2D ensemble-averaged mean-squared displacement of the polymer beads against time lag based on Equation (9) as follows:

(9) $$MSD_{2d}=4D_{xz}t^{\alpha }$$

where $MSD_{2d}$ is the averaged 2D mean-squared displacement of the polymer beads in cylindrical confinement, $D_{xz}$ is the ensemble-averaged 2D diffusion coefficient in the xz plane, *t* is the time lag, and α is the exponent characterizing the anomalous diffusion. And for fractional Brownian motion, α=2H. The averaged 2D MSD as a function of time lag for different Hurst parameters with both FENE and harmonic bonds are plotted in Figure 4a. Those MSDs show that the polymer beads connected by FENE bonds diffuse slightly faster than those connected by harmonic bonds, but this speeding-up effect is insignificant. One example of the exponential fitting of 2D MSD against time lag according to Equation (9) is presented in the inset of Figure 4b for H=0.50. It turns out that although the diffusions of polymer beads are affected by the neighboring beads through bonding energy as well as the excluded volume potentials, the diffusive behaviors of those polymer beads can still be well characterized by Equation (9). As shown in Figure 4b, the fitted diffusion coefficient of polymer beads increases as the Hurst parameter *H* increases, which is not surprising based on the fact that the larger the Hurst parameter *H*, the more correlated the fractional Gaussian noises are. Figure 4c demonstrates that the exponent α also increases as the Hurst parameter *H* increases for H∈(0.40,0.60). Previous experimental observations show that α for chromosomal loci is about 0.38∼0.40 [3,4,5], which agrees very well with the simulation results for H∈(0.45,0.50). This agreement indicates that the weak viscoelasticity of cytoplasm is sufficient for the subdiffusion of chromosomal loci in vivo. The fitted diffusion coefficient and exponent α also show that polymer beads connected by FENE bonds diffuse slightly faster than beads with harmonic bonds, especially for large Hurst parameters (H>0.50).

**Figure 5:**
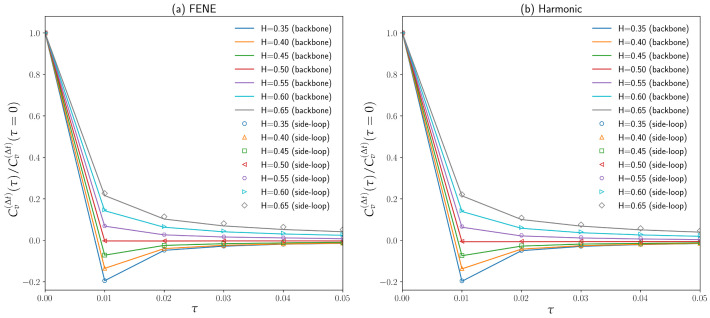
(**a**) Velocity autocorrelation function $C_{v}^{\left(\Delta t\right)}\left(\tau \right)$ of both a backbone chain bead and a side loop bead for different Hurst parameters with FENE bonding energy. (**b**) Velocity autocorrelation function $C_{v}^{\left(\Delta t\right)}\left(\tau \right)$ of both a backbone chain bead and a side loop bead for different Hurst parameters with harmonic bonds.

Although various random walks models such as CTRW [28], fBM [30], OD [27], and diffusing diffusivity [6] can reproduce anomalous subdiffusion, only fractional Brownian motion [29,30] seems to be promising concerning the anticorrelated velocity of chromosomal loci and cytoplasmic particles. The effects of the polymer model structures and excluded volume interactions between the polymer segments on the velocity autocorrelation function were examined in this work. The velocity autocorrelation function of a discrete-time process with a time step Δt is given by the equation below [3]:

(10) $$C_{v}^{\left(\Delta t\right)}\left(t\right)=\frac{1}{\Delta t^{2}}\langle \left(r(t+\Delta t)-r(t)\right)\cdot \left(r(\Delta t)-r(0)\right)\rangle$$

where r(t) is the bead position at time *t* and $C_{v}^{\left(\Delta t\right)}\left(t\right)$ is the velocity autocorrelation function with a time step Δt=0.01 at simulation time *t*. One polymer bead in the middle of the backbone chain (shown as the “backbone” in Figure 5) as well as one polymer bead on the side loop attached to the end bead of the backbone chain (shown as the “side-loop” in Figure 5) are chosen for the calculations of velocity autocorrelation functions. The normalized velocity autocorrelation functions $C_{v}^{\left(\Delta t\right)}\left(\tau \right)/C_{v}^{\left(\Delta t\right)}\left(\tau =0\right)$ are demonstrated in Figure 5 for polymer models with both FENE bonds and harmonic bonds. As can be seen in Figure 5, the velocity autocorrelation function reduces to 0 for H=0.5 within the first time step τ=Δt=0.01. It remains zero thereafter for ordinary Brownian motion (H=0.5) is an uncorrelated Markovian process. For small Hurst parameters (H<0.5), the velocity is anticorrelated, i.e., the velocity autocorrelation function has a negative part for τ>0, which agrees with previous experimental measurements of chromosomal loci [3,30]. As *H* increases from 0.35 to 0.50, this anticorrelation becomes weaker, resulting from a weaker “elasticity” of the cytoplasm. If the Hurst parameter *H* is above 0.50, the velocity of the polymer bead becomes correlated; that is, the velocity autocorrelation function remains positive for τ>0. Again, the polymer beads act as if they have “momenta” for H>0.50, although the masses of polymer beads are neglected in our simulations based on Equation (5).

**Figure 6:**
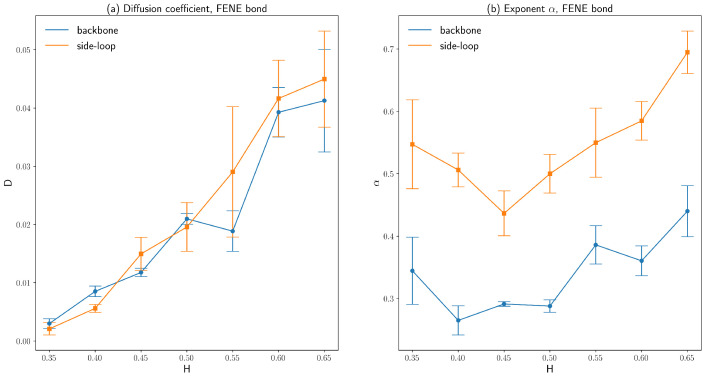
(**a**) The diffusion coefficients of a backbone chain bead and a side loop bead on a polymer model with FENE bonds for various Hurst parameters. (**b**) The exponent α characterizes anomalous diffusion of a backbone chain bead and a side loop bead on a polymer model with FENE bonds for various Hurst parameters (the error bars are standard deviations).

**Figure 7:**
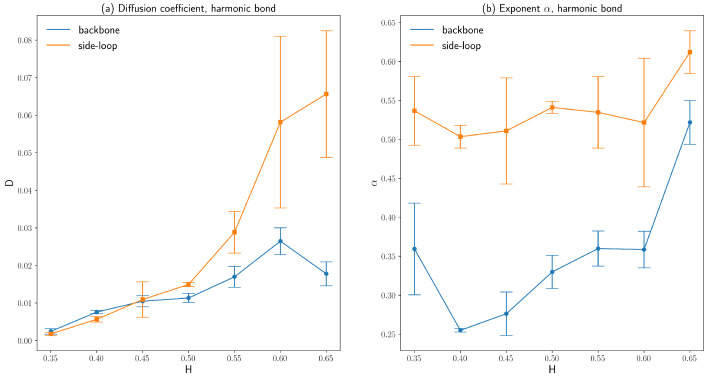
(**a**) The diffusion coefficients of a backbone chain bead and a side loop bead on a polymer model with harmonic bonds for various Hurst parameters. (**b**) The exponent α characterizing anomalous diffusion of a backbone chain bead and a side loop bead on a polymer model with harmonic bonds for various Hurst parameters (the error bars are standard deviations).

The diffusion coefficients, as well as the exponents α of those two polymer beads on the backbone chain and the side loop as described above, are obtained by averaging over three independent runs and are plotted as functions of Hurst parameters in Figure 6 and Figure 7. Similarly, the fitted diffusion coefficient of the polymer beads increases significantly as *H* increases, just like the fitted diffusion coefficient based on the ensemble-averaged MSD. The side loop polymer bead diffuses faster than the backbone polymer bead since the number density of the polymer beads around the backbone chain is larger than that around the side loop attached to the end of the backbone chain bead, which can be seen from Figure 3. Because the number density of the polymer beads is evenly distributed across the cell volume for small *H*, the error bars of the fitted diffusion coefficients are also small for H<0.5. The exponent α of the side loop polymer bead is close to 0.5, which agrees with the predictions by the simple Rouse model combined with the Langevin dynamics simulation [29]. However, the exponent α of the backbone polymer bead is 0.30∼0.35 for a small H≤0.5, owing to the interactions between the backbone polymer bead and the condensed polymer segments around the backbone chain.

By comparing Figure 6 and Figure 7, it can be concluded that the types of bonds connecting consecutive polymer beads have insignificant effects on the diffusive behaviors of polymer beads. Our simulation results show that polymer beads’ exponent α can differ depending on their positions. This variation in α agrees with previous experimental results [4,5].

### 3.3. Displacement Distributions of Polymer Beads with Fractional Brownian Noises

**Figure 8:**
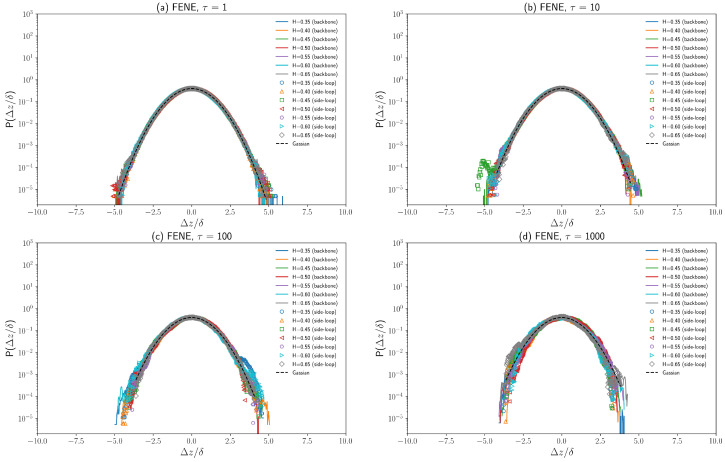
(**a**–**d**) One-dimensional displacement (Δz/δ) distributions of a backbone chain bead and a side loop bead on a polymer with FENE bonds for different Hurst parameters over different time lags (τ = 1∼1000). δ is the standard deviation of displacement in *z*-axis direction (Δz). The Gaussian distribution is plotted as a black dashed curve.

**Figure 9:**
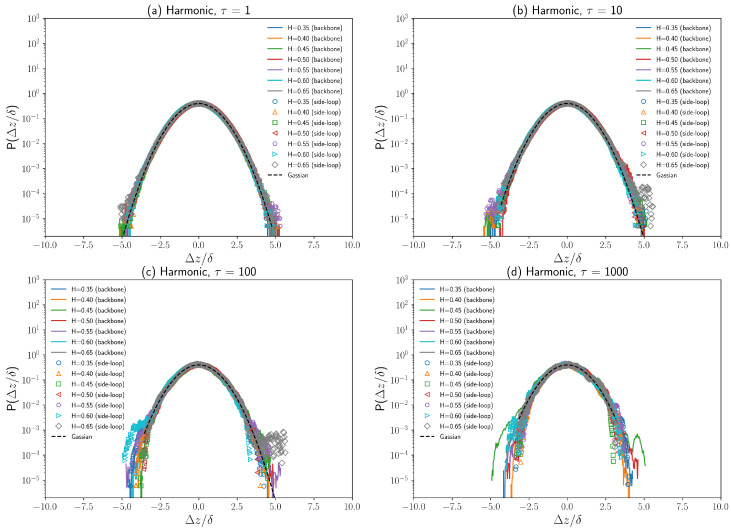
(**a**–**d**) One-dimensional displacement (Δz/δ) distributions of a backbone chain bead and a side loop bead on a polymer with harmonic bonds for different Hurst parameters over different time lags (τ = 1∼1000). δ is the standard deviation of displacement in *z*-axis direction (Δz). The Gaussian distribution is plotted as a black dashed curve.

Fractional Brownian motion is a Gaussian process with stationary increments [28,41]. So, the displacement distribution of a single tracer performing fractional Brownian motion is Gaussian. The major difference between the anomalous diffusive behavior of chromosomal loci [4,5] and cytoplasmic particles [6] is that chromosomal loci are affected not only by viscoelastic cytoplasm but also by interactions between DNA segments such as DNA bridging [25]. To investigate how the displacement distributions of polymer beads are affected by the viscoelasticity of cytoplasm as well as the polymer dynamical structure, the one-dimensional displacement distributions in the *z*-axis direction of both a backbone chain bead and a side loop bead are plotted in Figure 8 and Figure 9. Simulations show that the normalized 1D displacement (Δz/δ) distributions agree very well with normal distribution for different Hurst parameters *H*, as demonstrated in Figure 8 and Figure 9. This agreement indicates that polymer beads exhibit a non-Brownian yet Gaussian subdiffusion (for H≠0.5). The normalized 1D displacement distributions (Δz/δ) over different time lags (τ = 1, 10, 100, 1000) plotted in Figure 8 and Figure 9 show that the displacement distribution of both the backbone chain bead and side-side loop bead can remain Gaussian for a wide range of τ. However, the displacement distribution starts to deviate from the Gaussian distribution for large displacement (Δz>3δ), especially for τ=1000, as shown in Figure 9d. Such deviations can be attributed to insufficient sampling as well as cylindrical confinement. Moreover, the bonding energy (FENE/harmonic) has negligible effects on displacement distributions, which agrees with our previous analysis on diffusion coefficients and exponent α.

Recent experimental results show that the displacement distributions of cytoplasmic particles [6] as well as chromosomal loci [44] follow a Laplace distribution. Many mathematical combinations of random variables can produce the Laplace random variable. A straightforward example is that the Laplace random variable can be represented by the product of a Gaussian random variable and the square root of an exponential random variable. Moreover, RNA-protein particle diffusivities exhibit an exponential distribution [6], which might contribute to the Laplace distribution of cytoplasmic particle displacement. Chubynsky [33] developed a diffusing diffusivity model that results in an exponential diffusivity distribution for specific conditions. Hence, the diffusing diffusivity model has attracted more attention recently [32] as it has great potential to elucidate the origin of the non-Gaussian behaviors of cytoplasmic particles and chromosomal loci. However, the simple version of the diffusing diffusivity model cannot explain the anticorrelated velocity of tracers in the cytoplasm, as such a simple model does not have a “memory” as the fractional Brownian motion model does. Using fractional Gaussian noise for each polymer bead during our fractional Brownian dynamics simulations, we implicitly assume that the viscoelasticity of cytoplasm remains unchanged across the cell, which is not true as the cytoplasm is a heterogeneous environment [35]. It is possible as well as reasonable to combine the fractional Brownian dynamics as described above with a random diffusivity of the chromosomal locus to model the non-Brownian and non-Gaussian subdiffusion in vivo [6,44]. One possible method to include diffusing or random diffusivity into this fractional Brownian dynamics simulation is assigning a different radius $a_{bead}$ to polymer beads in different areas within the cylindrical confinement to simulate the heterogeneity of the interior environment of the bacterial cell.

In this article, in order to simulate the anomalous diffusion of chromosomal loci, we use fractional Gaussian noise instead of white noise (the Brownian force) to simulate the effects of viscoelastic cytoplasm on polymer segments to reduce the number of degrees of freedom of the simulation system. This implicit model of cytoplasm speeds up simulations dramatically by averaging over nonessential degrees of freedom. However, the significant reduction in degrees of freedom obstructs the accurate computation of system entropy. The crowding effect, which is crucial in chromosome organization [45,46], cannot be studied directly in our simple model. Furthermore, since fractional Gaussian noise is highly correlated, generating fractional Gaussian noise is much more complex than generating ordinary Gaussian random numbers. For the simulation results presented in this manuscript, the Hurst parameter *H* is varied only between 0.35 and 0.65, mainly due to the fact that generating fractional Gaussian noise becomes extremely slow for a small H<0.35 and large H>0.65. The fBm-0.3.0 package we used for fGn generation switches from the fast Davies Harte method [42] to the slow Hosking algorithm [41] for small and large Hurst parameters. In addition, the fGn generation has to be carried out at the beginning of fractional Brownian dynamics simulations, which is computationally expensive. In contrast, the white noise generation in ordinary Brownian dynamics simulations can be performed on the fly. So, it is not easy to apply this fractional Brownian dynamics method to larger systems, for example, a modified version of this “bottle-brush”-type polymer model of bacterial chromosomes developed by Swain et al. [21] which contains about 40,000 polymer beads and extra crowders.

Since chromosomal loci perform subdiffusion in cells, it seems less attractive to carry out fractional Brownian dynamics simulations for a large H>0.50, where fractional Brownian particles perform accelerated Brownian motion. However, as the nonequilibrium metabolic activities play a crucial role in cytoplasmic subdiffusion and can be modeled by a random force [37], the diffusion of polymer beads might be simulated by an overlay of an fGn with H<0.5 and an fGn with H>0.50, where both viscoelastic cytoplasm and metabolic activity are modeled by fractional Gaussian noise. Nevertheless, mapping the random forces exerted on polymer segments to the metabolic activities that occur in cells is highly challenging.

Another problem of this fractional Brownian dynamics simulation is the sufficient sample size. Although ergodicity is not broken within fractional Brownian motion [28], fBm is a self-similar process [41]. As a result, for a large *H*, fBm is ballistic-motion-like, as shown by the green curve in Figure 1b. To obtain the accurate ensemble-averaged diffusivity, a large sample size is required for fractional Brownian motion with a large *H* compared to a standard Brownian dynamics simulation since analytical solutions to accelerated Brownian motion are only available for one-dimensional problems [38]. This sample size problem also explains the large standard deviations of the fitted diffusion coefficients for H>0.55, as shown in Figure 6 and Figure 7.

## 4. Conclusions

Using a simple “bottle-brush”-type polymer model combined with fractional Gaussian noises, the anomalous diffusive behaviors of polymer segments in viscoelastic fluid are investigated systematically by using a homemade fractional Brownian dynamics simulation code. The standard Brownian dynamics simulations are carried out by setting the Hurst parameter *H* to 0.5. Moreover, the standard Brownian dynamics simulation results are compared to previous experimental and theoretical studies to verify the fractional Brownian dynamics code. By varying the Hurst parameter *H*, the dynamical structures and the anomalous diffusivity of the polymer model are explored, from a subdiffusive regime to a superdiffusive regime. Our fractional Brownian dynamics simulation demonstrates that both the subdiffusive behaviors of polymer beads (α=0.38∼0.40) and the anticorrelated velocity of chromosomal loci can be attributed to the weak viscoelasticity (H≈0.45) of cytoplasm. It is crude to assume that fractional Gaussian noises for different polymer beads are independent ($EX_{i}^{\left(n+k\right)}X_{j}^{\left(n\right)}=0$ for i≠j) since polymer beads that are close to each other have a similar environment and thus experience correlated fractional Gaussian noises ($EX_{i}^{\left(n+k\right)}X_{j}^{\left(n\right)}\neq 0$ for small $|r_{i}-r_{j}|$, k≈0). However, our assumption of an independent fGn might be sufficient for the simulations of the subdiffusion of chromosomal loci because the exponent α=0.38∼0.40 characterizing the anomalous subdiffusion of chromosomal loci can be reproduced with a Hurst parameter H∈(0.45,0.50), where the autocorrelation function of fGn (Equation (8)) decays rapidly. Since our simulations do not include the heterogeneity of cytoplasm and metabolic activity, this simple model does not reproduce the Laplace displacement distribution of cytoplasmic anomalous diffusion. Nevertheless, this fractional Brownian dynamics simulation method can be used to investigate many crucial biological reactions, such as chromosome segregation, facilitated diffusion [39], and the responses of bacterial chromosomes to external mechanical forces [47], as the viscoelasticity of cytoplasm plays a vital role in such processes.

## Data Availability

Data are contained within the article.
